# Clinical Investigations into Diagnosing and Managing Strabismus: From Etiological Diversity to Personalized Therapeutic Strategies

**DOI:** 10.3390/jcm15051915

**Published:** 2026-03-03

**Authors:** Mario Cantó-Cerdán, Pilar Cacho-Martínez

**Affiliations:** 1 VISSUM, Miranza Group, C/Cabañal 1, 03016 Alicante, Spain; 2 Grupo de Investigación en Optometría (GIOptom), Universidad de Alicante, 03690 Alicante, Spain; cacho@ua.es; 3 Departamento de Óptica, Farmacología y Anatomía, Universidad de Alicante, 03690 Alicante, Spain

Strabismus is a complex disorder of binocular vision characterized not only by ocular misalignment but also by disturbances in sensory fusion, stereopsis, and ocular motor coordination. It affects individuals across the lifespan and may lead to diplopia, reduced depth perception, visual discomfort, and significant psychosocial consequences. Beyond functional impairment, strabismus has been associated with a diminished quality of life, social stigma, and mental health challenges, emphasizing the importance of effective management strategies [1,2]. Despite advances in surgical techniques and diagnostic tools, optimal care remains challenging due to the heterogeneity of etiologies and the multifactorial determinants of treatment outcomes [3,4].

This Special Issue, “Clinical Investigations into Diagnosing and Managing Strabismus”, presents a diverse collection of studies that reflect the contemporary landscape of strabismus research and clinical care. The contributions span a wide range of underlying conditions, including acquired forms in otherwise healthy adults, neurologic and mechanical etiologies, genetic disorders affecting visual development, and emerging rehabilitative interventions. Collectively, they emphasize a paradigm shift toward individualized etiology-driven management strategies that address both motor alignment and functional vision.

One major theme emerging from this Special Issue is the diversity of clinical presentations and mechanisms underlying strabismus. Adult-acquired esotropia, for instance, poses diagnostic and therapeutic challenges because of its insidious onset and previously normal binocular vision. A retrospective study included in this Special Issue describes a subgroup characterized by distance-predominant diplopia, moderate refractive error, and high fusional amplitudes, suggesting a distinct clinical entity not fully captured by traditional classifications. Treatment combining prism correction and lateral rectus resection produced favorable sensory and motor outcomes, underscoring the importance of tailoring therapy to functional characteristics rather than the deviation magnitude alone [5].

The role of the deviation type and ocular motor behavior in determining surgical outcomes is further explored in a prospective study comparing esotropia and exotropia. Using video-oculography to objectively assess alignment across multiple gaze positions, the authors reported significant postoperative improvements in both conditions, with the highest efficacy observed in the primary position of gaze. However, alignment in non-primary positions was less predictable, highlighting the limitations of traditional assessment methods that focus solely on primary gaze measurements. These findings support the incorporation of objective multigaze evaluation into routine clinical practice to better characterize the functional outcomes following surgery [6].

Several studies in this Special Issue address strabismus secondary to specific pathologies, emphasizing the importance of etiological diagnosis. Mechanical strabismus following functional endoscopic sinus surgery represents a rare but severe complication caused by extraocular muscle injury or orbital damage. A case series demonstrates that early recognition and prompt surgical repair are critical for achieving satisfactory alignment and eliminating diplopia, whereas delayed intervention often leads to persistent motility deficits and the need for multiple procedures [7]. These findings highlight the need for interdisciplinary collaboration between otolaryngologists and ophthalmologists in managing orbital complications.

Paralytic strabismus due to cranial nerve palsy constitutes another challenging clinical scenario. A large retrospective analysis included in this Special Issue reported significant postoperative reductions in the angle of deviation and satisfactory outcomes in most patients, regardless of the demographic factors or etiology. Nevertheless, surgical planning in these cases remains complex, due to incomitant deviations and residual muscle dysfunction, often requiring individualized procedures targeting multiple muscles [8].

Beyond acquired and mechanical causes, the issue also addresses visual function in systemic and neurodevelopmental disorders. In children with STXBP1-related epileptic encephalopathy, high rates of refractive error, accommodative deficits, and abnormal binocular vision were observed despite the absence of major structural ocular abnormalities. These findings suggest that neural dysfunction alone can profoundly disrupt visual development and support the need for early ophthalmologic evaluation and intervention in patients with genetic neurological conditions [9].

Although surgical correction remains central to treatment, the restoration of binocular function increasingly relies on complementary non-surgical approaches. A pilot study presented in this Special Issue investigated a virtual reality-based perceptual learning protocol aimed at improving stereopsis in adults with severe stereodeficiency. The participants demonstrated gains in stereoacuity, fusional vergence, and binocular function after a short intervention period, with some achieving measurable global stereopsis for the first time. These results, while preliminary, suggest that immersive digital technologies may promote neural plasticity and functional recovery even in adulthood [10].

Taken together, the contributions in this Special Issue reflect a transition from a purely motor-centric view of strabismus toward a comprehensive framework integrating sensory function, neural mechanisms, and patient-specific factors. Accurate diagnosis now requires not only the measurement of ocular deviation but also the assessment of binocular vision, ocular motility patterns, refractive status, and associated systemic or neurological conditions. Likewise, the treatment success should be evaluated in terms of functional outcomes such as diplopia resolution, stereopsis, and quality of life, rather than alignment alone.

Despite these advances, important gaps remain. Standardized outcome measures are lacking, limiting comparability across studies. Objective technologies such as eye tracking and video-based quantification show promise for improving alignment assessment across gaze positions and for enabling more automated reproducible measurements [11,12,13]. Furthermore, long-term evidence regarding novel rehabilitative strategies, including virtual reality-based therapies, remains limited. Understanding the mechanisms underlying the decompensation of previously controlled deviations, particularly in adults, also warrants further investigation, including clinical scenarios of recently acquired diplopia in adults with a history of longstanding strabismus [14].

Emerging technologies may help address these challenges. Artificial intelligence and machine learning have the potential to improve the diagnostic accuracy, predict treatment outcomes, and support personalized management strategies. Recent bibliometric analyses suggest a rapidly growing body of work in AI applied to strabismus detection and measurement, with “deep learning” and “machine learning” emerging as major hotspots [13]. Advances in digital health platforms may enable remote monitoring and tele-rehabilitation, expanding access to specialized care. However, the integration of these tools must remain grounded in clinical expertise and patient-centered considerations.

In conclusion, the studies assembled in this Special Issue highlight the multifaceted nature of strabismus and the need for personalized approaches tailored to each patient’s underlying condition and functional goals. By integrating etiological insights, surgical innovations, and novel rehabilitative strategies, these contributions advance our understanding of how to diagnose and manage this complex disorder more effectively. Continued interdisciplinary collaboration and rigorous clinical research will be essential to translate these advances into improved outcomes for patients with strabismus across the lifespan.
